# Indirect Restorations and Fixed Prosthodontics: Materials and Techniques Used by General Dentists of New Zealand

**DOI:** 10.1155/2019/5210162

**Published:** 2019-01-10

**Authors:** Paul A. Brunton, Jithendra Ratnayake, Carolina Loch, Arthi Veerasamy, Peter Cathro, Robert Lee

**Affiliations:** Faculty of Dentistry, University of Otago, 310 Great King Street, Dunedin 9016, New Zealand

## Abstract

**Background:**

To investigate the selection and use of materials and techniques for core buildup, indirect restorations, and fixed prosthodontics by general dentists in New Zealand.

**Methods:**

A questionnaire comprising 19 sections and 125 questions was distributed via mail to 351 general dentists in New Zealand who were selected from the Dental Council of New Zealand's 2016 register.

**Results:**

The majority of the respondents (68.8%) reported using resin composite light-cured materials for the core buildup of vital posterior teeth. A large number of respondents (52%) did not use dentine pins, with the majority of them (25%) being recent graduates (<10 years). Fibre posts were used by 61.6% of the dentists surveyed. The majority of dentists (54.6%) reported using addition-cured silicone impression material for crown and bridge impressions. Glass-ionomer cements (37.5% of participants) and resin-modified glass-ionomer cements (35.8%) were the most common luting cements used. Direct resin composite veneers were the preferred material of choice rather than indirect restoration of anterior teeth (40.4%).

**Conclusions:**

The study showed that New Zealand dentists surveyed are using current state-of-the-art materials and techniques, with their choice of material being greatly influenced by clinical indications and patients aesthetic demands.

## 1. Introduction

Oral health is an integral part of general health, and good oral health is important for overall quality of life [1, 2]. However, oral health diseases such as dental caries, tooth loss, periodontal diseases, and oral cancer are still a major public health concern in the world. Risk factors associated with oral diseases include unhealthy diets, tobacco and harmful alcohol use, and poor oral hygiene [1]. Dental caries is a global disease affecting all ages and sectors of the population. Despite the advancement in early detection and treatment, it remains as one the most prevalent chronic diseases in the world [3, 4]. Poor oral health affects people both physically and psychologically and influences their social well-being. With the increasing ageing population, most people are retaining their natural dentition for longer [5]. In addition, the increasing media coverage of dental and oral issues has increased public awareness of the benefits of good oral health and the role of aesthetics.

At present, dentistry is a highly commercialised profession, which regularly faces the introduction of new technologies, techniques, and materials. In general, patients presenting with missing teeth would like to have their teeth replaced with the most aesthetically appealing and long-lasting material and technique possible. This should also take into account maximum preservation of sound tooth structure, avoidance of removable prostheses whenever possible, minimal surgical risk, as well as cost-effectiveness and a low-maintenance design [6, 7]. Noticeable changes in the use of restorative materials have occurred during the past decade due to aesthetic considerations [8–10]. The introduction of new and refined restorative materials and techniques, changes in restorative treatment patterns, and effective preventive programs have greatly influenced the longevity of dental restorations. In addition, there is a growing concern about the use of metallic alloys in particular amalgam, due to alleged health effects and environmental consideration [11, 12]. Therefore, more aesthetically appealing restorations tend to predominate in contemporary dentistry.

Given the ever-increasing rate of change in contemporary clinical dentistry, it is important to investigate the treatment options and choice of materials by general dentists. The primary aim of this study was to investigate the selection and the use of materials and techniques for core buildups, indirect restorations, and fixed prosthodontics by general dentists in New Zealand.

## 2. Materials and Methods

Ethical approval for this research was obtained from the University of Otago Human Ethics committee (approval number D16/098). A questionnaire comprising 19 sections and 125 questions was distributed to a sample of 351 dentists who were selected from the 2016 Dental Council of New Zealand's register. A stratified sampling procedure was done proportionally to the number of dentists registered in each NZ region. The questionnaire was sent together with a cover letter, addressed return envelope, and $5 coffee voucher. After four weeks, an e-mail reminder was sent to all the participants who did not respond (a detailed description of the Materials section is given in Lee et al. [14] (the Methods section described in Lee et al. is attached as an appendix for reviewers of this manuscript)).

The following topics were investigated in relation to the provision of indirect restoration and fixed prosthodontics, which was similar to the topics covered in a previous UK-based study [13]:

- Material selection for core buildup on vital teeth
- The types of post and core systems used
- Impression materials, alloys, and luting cements used
- Preference for full or partial coverage restorations
- Use of metal-free restorations

The data from the returned questionnaire were weighted proportionally to correct the potential survey bias to adjust the difference in representativity between New Zealand regions. The data were analysed using Statistical Package for Social Studies software (SPSS version 24; IBM Corporation, Armonk, NY, USA). Bivariate analyses were conducted using the chi-squared test to test the association between materials and techniques used for procedures and the following demographic variables: years since graduation, gender, and practice location. The level of significance was set at *p* < 0.05.

## 3. Results

From the 351 questionnaires sent, a total of 204 completed questionnaires were returned, giving an overall response rate of 58%. After checking the validity and completeness of the returned questionnaires, only 188 questionnaires were deemed usable. The demographic details of the respondents have already been described [14].

### 3.1. Core Buildup for Vital Teeth

The majority of respondents (*n* = 129; 68.8%) reported using light-cured resin composite as their preferred material of choice for the core buildup of vital posterior teeth, with amalgam (*n* = 77; 40.6%) and resin composite dual-cured materials (*n* = 68; 36.5%) as preferred alternatives (Table 1). There was a statistically significant association between the time since a respondent had graduated and those who preferred light-cured resin composites (*X* ^2^ = 21.139; *p* < 0.005). The majority of the dentists who reported using light-cured resin composites were recent graduates (*n* = 32; 24.6%) in comparison to the dentists who graduated 40 or more years ago (*n* = 10; 18.3%).

### 3.2. Dentine Pins

**Figure 1:**
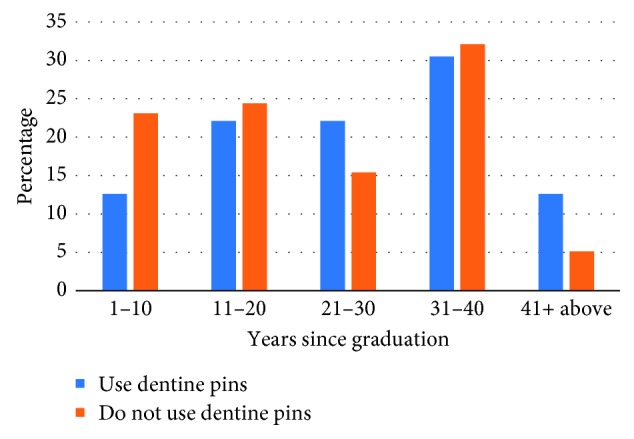
Association between years since graduation and use of dentine pins.

More than half of the respondents indicated that they do not use dentine pins (*n* = 98; 52%). The majority of the dentists who use dentine pins graduated 31 or more years ago (*n* = 38; 42.6%), whereas the use of dentine pins among recent graduates (graduated <10 years) was less common (*n* = 12; 12.6%). This association was found to be statistically significant (*X* ^2^ = 9.809; *p* < 0.05) (Figure 1).

### 3.3. Post Systems

Fibre posts (*n* = 113; 61.6%) were preferred by most dentists in this study, followed by stainless steel (*n* = 60; 32.6%) and indirect cast posts from either a precious (*n* = 50; 26.3%) or nonprecious alloy (*n* = 40; 21.7%). Less frequently used post materials were pure titanium (*n* = 10; 4.8%) and titanium alloys (*n* = 28, 14.6%). A small proportion of dentists reported that they do not use fibre posts in their practice (*n* = 16; 8.3%) (Table 2).

There was no significant association found between the use of fibre post systems and gender, years since graduation, and practice location. However, when comparing suburban and rural dentists, a considerable number of urban dentists (*n* = 87; 72.5%) reported not using stainless steel posts and core systems and this result was statistically significant (*X* ^2^ = 7.365; *p* < 0.05).

### 3.4. Impression Materials

Addition-cured silicone (*n* = 105; 54.6%) and polyether impression materials (*n* = 56; 30.9%) were the most common impression materials used. Impression materials less frequently used were condensation cured silicone (*n* = 10; 5.3%) and alginate (*n* = 7; 4%) (Table 3). A statistically significant association was found between the use of addition-cured silicone and practice location (*X* ^2^ = 9.480; *p* < 0.05). The addition-cured silicone impression material was preferred among urban dentists in comparison to suburban and rural dentists.

**Figure 2:**
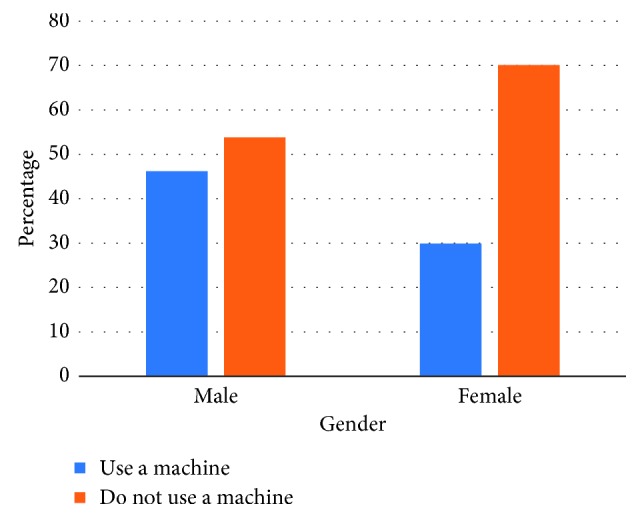
Association between gender and use of an automatic impression mixing machine.

Nearly 60% (*n* = 112) of the dentists reported using an automatic impression mixing machine. There was a statistically significant association between the use of an automatic impression mixing machine and gender (*X* ^2^ = 5.331; *p* < 0.05). Male dentists used the mixing machine more frequently (*n* = 80; 46.2%) compared to female dentists (*n* = 32; 29.9%) (Figure 2).

### 3.5. Alloys Used for Fixed Prosthodontics

The majority of the dentists in this study reported using precious alloys (*n* = 93; 48.6%), followed by nonprecious alloys (*n* = 52; 27.7%), for fixed prosthodontics. A considerable number of participants reported using a combination of both precious and nonprecious alloys (*n* = 10; 5.4%) and also semiprecious alloys (*n* = 15; 8.5%) (Table 4).

### 3.6. Luting Cements

Luting cements based on resin-modified glass-ionomer cements were used to cement single zirconia units by the majority of the dentists in this study (*n* = 34; 18.7%). Resin composite-based luting cements and self-adhesive resin cements were the second most used luting cements. Traditional glass-ionomer (*n* = 20; 11.4%) and resin-based cements (*n* = 16; 7.7%) were used by fewer dentists (Table 5). A statistically significant association was found between the use of self-adhesive luting cement and practice location (*X* ^2^ = 7.436; *p* < 0.05), where urban dentists used self-adhesive luting cements more frequently compared to suburban and rural dentists.

For porcelain-fused-to-metal reconstructions, luting cements based on glass ionomers (*n* = 70; 37.5%) and resin-modified glass ionomers (*n* = 66; 35.8%) were commonly used amongst the respondents to the survey. Resin composite (*n* = 28; 14.5%) and self-adhesive cements, which adhere specifically to metals (*n* = 25; 13.2%), were more frequently used in comparison with resin-based cements (*n* = 19; 8.7%).

### 3.7. Choice of Indirect Restoration for Anterior Teeth

The preferred material of choice for restoring anterior teeth was direct resin composite veneers (*n* = 77; 40.4%). Some dentists (*n* = 55; 29.8%), however, still favoured the use of laboratory-fabricated porcelain veneers. About 12% (*n* = 22) of dentists reported using both direct resin composite and laboratory-made porcelain veneers depending on the patients' needs. Less than 3% of dentists reported using CAD-CAM milled veneers, and 9% (*n* = 6) did not prescribe veneers for their patients.

### 3.8. Use of Tooth-Coloured Inlays/Onlays and Metal-Free Crowns

Ceramic (*n* = 108; 57.7%) was the most preferred material for tooth-coloured inlays and onlays for posterior teeth, followed by composite resins (*n* = 27; 14%). No significant association was found between the choice of tooth-coloured inlay/onlay materials for posterior teeth and the selected demographic variables. More than half of the dentists surveyed (57%, *n* = 105) routinely provided tooth-coloured metal-free crowns for their patients, whilst 31.1% (*n* = 60) provided them occasionally. Some dentists never provided metal-free crowns (8.8%, *n* = 16).

## 4. Discussion

The present study provides valuable information on choice of the materials for core buildups, indirect restorations, and fixed prosthodontics by general dentists in New Zealand. The response rate of 58% is such that results can be interpreted with reasonable confidence. Studies such as this provide a valuable insight into the general dental practice in New Zealand. In addition, it provides the opportunity to collect further baseline data on the New Zealand dental workforce, enabling future comparisons over time and with other countries around the world.

Light-cured resin composite was the preferred material for core buildup of vital teeth in NZ. This was in stark contrast to the UK survey in 2008, where amalgam (65%) was the preferred material due to its longevity [15]. In a survey conducted in Australia to assess the attitudes and preferences of Australian prosthodontists for post usage in endodontically treated teeth, the majority of the respondents reported that dual-cured resin composite was the most popular core material (34%) for prefabricated posts, followed by light-cured resin composite (29%) and amalgam (28%). Resin-modified GIC and GIC were the least used core materials, at only 2% each [16]. Although dental amalgam is one of the most versatile restorative materials used in dentistry, mainly due to its durability and technique insensitivity, there is still continuing debate over its safety and efficacy [11]. Following the Minamata convention, which obliged countries to minimize the anthropogenic emission of mercury and its products, the use of amalgam has phased down in many countries, and this may explain the difference noted in this study [17]. This survey showed that among the dentists sampled in NZ, light-cured resin composite was favoured over amalgam for the core buildup of vital teeth. This comes as no surprise given the superior physical, mechanical, and aesthetic properties of resin composite materials, which include closely mimicking the natural tooth structure, greater versatility, and reparability [18]. Dentists who graduated less than 10 years ago (24.6%) preferred the use of light-cured resin composites compared to dentists who graduated more than 30 years ago. This difference, which was statistically significant (*p* < 0.05), might reflect the more up-to-date teaching methods of undergraduate courses in New Zealand, which are evolving to incorporate evidence-based mainstream general dental practice.

The declining use of dentine pins was observed in this study, which was also consistent with the previous UK investigation [13]. This could be mainly due to the well-documented disadvantages associated with dentine pins such as microleakage, dentinal microfractures, and lowered fracture resistance [19, 20]. Recent graduates (graduated <10 years) used less dentine pins compared to older graduates. This possibly reflects changes in undergraduate teaching, which has been driven by evidence-based practice. However, further research should be conducted to investigate the dynamics and driving factors behind such changes.

In contrast to a previous study in the UK where the use of indirect casts of precious alloys predominated [13], fibre posts were significantly used by dentists in New Zealand (61.6%). This suggests a move away from traditional post systems based on metals such as precious and nonprecious alloys (e.g., stainless steel and titanium). Fibre posts are a relatively new advancement in dentistry and have gained popularity in the dental market over the past decade [21]. It is evident from this survey that dentists in New Zealand have recognised the considerable benefits of fibre posts which include superior physical properties (modulus of elasticity, compressive strength, flexural strength, and thermal expansion similar to that of dentin), ease in manipulation, better aesthetics (natural translucency to the dentine), and removability [22]. However, in Australia, the majority of dentists of a previous study indicated a preference for custom cast metal posts for endontically treated teeth. This was followed by prefabricated metal and prefabricated fibre-reinforced (FRC) posts [16]. In a future study, it would be valuable to investigate what are the preferred types of fibre post systems used and whether there would be significant advantages over other systems.

Addition-cured silicone impression materials were used by the majority of the dentists who responded to the survey. This should come as no surprise since it is considered as the most dimensionally stable impression material and exhibits pseudoplastic properties where it can be used as either syringe or tray material [23]. The use of addition-cured silicone was significantly higher among urban dentists compared to both suburban and rural dentists. This raises the question whether cost and availability of the material are the main barriers for suburban and rural dentists when using addition-cured silicone as an impression material. A large proportion of dentists (31%) indicated that they use polyether impression materials. This might be because of the fast setting time (<5 minutes) of polyether. However, it requires considerable force to remove the impression from both the patient's mouth and also from the stone cast when set [23]. Condensation-cured silicone and alginate impression materials were the least preferred materials, and this could be due their poor dimensional stability. The majority of respondents (60%) reported using an automatic impression mixing machine, which suggests that dentists are embracing new advancements in dental technologies and techniques centred around impression making [24]. No such survey has been conducted in Australia, to date, to investigate the types of impression material used in general dental practice. Future studies should also investigate the availability and use of intraoral scanners for digital impressions in general dental practice.

When comparing the use of luting cements with a previous UK study [13], two striking differences were found. Firstly, zinc phosphate cements were still used by a significant number of dentists in the UK (∼28%), whereas in New Zealand, less than 1% used zinc phosphate luting cements for most porcelain-fused-to-metal restorations. In addition, luting cements based on glass-ionomer and resin-modified glass-ionomer technology were frequently used by dentists in New Zealand. Secondly, recently developed luting cements based on resin-modified glass ionomers to cement single zirconia units were used by the majority of dentists in New Zealand, whilst their UK counterparts preferred the use of traditional glass-ionomer cements [13]. A similar finding was observed in Australia, with resin composite being the most frequently used luting cement, followed by both resin-modified glass-ionomer cement and glass-ionomer cement. However, zinc phosphate cement was still used by 11% of the dentists in Australia [16]. This illustrates that the dentists in New Zealand who responded the survey are adopting newer luting cements with superior properties than more conventional materials.

Veneers were still the preferred choice for the restoration of anterior teeth, which is in accordance with the UK study [13]. The majority of dentists (40.4%) preferred direct resin composite veneers, although a few dentists (29.8%) reported using laboratory-fabricated porcelain veneers. This is to be expected given the number of advantages of direct resin composite veneers such as low cost, better aesthetics, and reversibility [25, 26]. Less than 3% of the dentists reported making CAD/CAM-milled veneers. This could be explained by the fact that CAD/CAM-milled veneers require a lot of time, special software, and technical expertise [27, 28]. Surprisingly, 9% of the dentists did not prescribe veneers to their patients, and it would be interesting to investigate the reasons behind this approach to patient care.

The use of ceramic crowns was popular amongst dentists in New Zealand, which was similar to a previous survey from the UK [13]. Surprisingly, only a minority of the dentists (14%) used composites for tooth-coloured inlays/onlays in New Zealand considering their excellent aesthetic and physical properties [29, 30]. A larger proportion of respondents indicated that they provided metal-free crowns on a routine basis, and this may be due to patients' desire (e.g., better aesthetics) of having metal-free crowns.

Although the materials and techniques used for indirect restorations and fixed prosthodontics by NZ dentists seem to be similar to those used in the UK, dentists in New Zealand who participated in this survey appear to be more rapidly adopting newer materials, technologies, and techniques to provide high quality evidence-based treatment to their patients. It is suggested that further research is needed to further understand current trends among dentists and clinical practice.

## 5. Conclusions

It is important to acknowledge that studies such as this one have a number of limitations. Data obtained in the current study are related to dental practitioners in New Zealand who responded to the study; however, findings and conclusions reported here can be applicable to other countries with similar practicing arrangements. The study suggests that New Zealand dentists are adopting current techniques and materials available in dentistry such as fibre posts, automatic impression mixing machines, resin-modified GIC, zirconia single units, and resin composite veneers. Even though the majority of them are using the latest techniques and materials, and supplying evidence-based care to the patients, the location of the practice and dentists' year of graduation seem to have a significant impact on their preferences.

## Figures and Tables

**Table 1:** Materials used for core buildup of vital teeth among New Zealand dentists surveyed.

Material	Frequency (*n*)	Weighted percent (%)
Resin composite light-cured	129	68.8
Amalgam	77	40.6
Resin composite dual-cured	68	36.5
Resin-modified glass-ionomer cement	22	11.6
Resin-modified glass ionomer	31	17.3
Reinforced glass-ionomer cement	17	9.1
Traditional glass-ionomer cement	14	7.1
Compomer	7	3.6
Do not place crown buildups	4	2.1
Other (bonded EMAX, Fuji IX)	7	3.9

**Table 2:** Type of post systems used by respondents.

Post used	Frequency (*n*)	Weighted percent (%)
Fibre posts	113	61.6
Stainless steel	60	32.6
Cast, precious	50	26.3
Cast, nonprecious	40	21.7
Titanium alloy	28	14.6
Titanium, pure	10	4.8
Do not place posts	16	8.3

**Table 3:** Type of impression materials used by the dentists surveyed.

Impression material	Frequency (*n*)	Weighted percent (%)
Addition-cured silicone	105	54.6
Polyether	56	30.9
Condensation cured silicone	10	5.2
Alginate	7	4
Other (*reaction silicone, aquasil, impregum, omnicam*)	14	7.6

**Table 4:** Type of alloys used by the respondents for fixed prosthodontics.

Alloy	Actual frequency (*n*)	Weighted percent (%)
Precious	93	48.6
Nonprecious	52	27.7
Both	10	5.4
Semiprecious	15	8.5
Do not use, not applicable	18	9.8

**Table 5:** Type of luting cements used to cement single zirconia units.

Post used	Frequency (*n*)	Weighted percent (%)
Resin-modified glass-ionomer cement	38	20.2
Resin composite-based luting cement	34	18.7
Self-adhesive resin cement	30	15.9
Glass-ionomer cement	20	11.4
Resin-based cement	16	7.7
Zinc phosphate	1	0.4
Others^*∗*^ (dual cure, speed CEM)	5	2.8

## Data Availability

The data used to support the findings of this study are available from the corresponding author upon request.

## Supplementary Materials

Supplementary Materials A detailed description of the methods is attached. Refer the Methods section in the manuscript.
